# Validation of the Organizational Culture Assessment Instrument

**DOI:** 10.1371/journal.pone.0092879

**Published:** 2014-03-25

**Authors:** Brody Heritage, Clare Pollock, Lynne Roberts

**Affiliations:** 1 School of Psychology and Speech Pathology, Curtin University, Perth, Western Australia, Australia; 2 Office of the Deputy Vice Chancellor Academic, Curtin University, Perth, Western Australia, Australia; University of Stellenbosch, South Africa

## Abstract

Organizational culture is a commonly studied area in industrial/organizational psychology due to its important role in workplace behaviour, cognitions, and outcomes. Jung et al.'s [1] review of the psychometric properties of organizational culture measurement instruments noted many instruments have limited validation data despite frequent use in both theoretical and applied situations. The Organizational Culture Assessment Instrument (OCAI) has had conflicting data regarding its psychometric properties, particularly regarding its factor structure. Our study examined the factor structure and criterion validity of the OCAI using robust analysis methods on data gathered from 328 (females = 226, males = 102) Australian employees. Confirmatory factor analysis supported a four factor structure of the OCAI for both ideal and current organizational culture perspectives. Current organizational culture data demonstrated expected reciprocally-opposed relationships between three of the four OCAI factors and the outcome variable of job satisfaction but ideal culture data did not, thus indicating possible weak criterion validity when the OCAI is used to assess ideal culture. Based on the mixed evidence regarding the measure's properties, further examination of the factor structure and broad validity of the measure is encouraged.

## Introduction

Organizational culture is an important construct within the I/O psychology literature, reflected in the multitude of conceptualisations and measurement approaches, and consistently reported associations with organizationally-relevant outcomes [2]–[6]. While organizational culture is often examined from the perspective of person-organization fit [2], [7], demonstrated links between perceptions of organizational culture and organizational outcomes such as organizational effectiveness [8], [9] form an important proportion of the literature relevant to this construct. A key issue in examining the veracity of these links between the construct of culture and organizational outcomes is validation of the means by which data is collected, and whether this data is representative of the latent constructs or observable phenomena being investigated. This primary interest in establishing validity, or whether measurement is approximating with sufficient accuracy the true relationships between variables [10], is of particular importance when establishing the theoretical properties of organizational culture. Similarly, as practitioners within the field seek to establish accurate measurement of unobservable phenomena (e.g., organizational culture as a prelude to organizational change), understandably the tools used by the field must be capable of delivering on this requirement. To this end, it is troubling that the evidence substantiating the validity of instruments used to measure organizational culture is limited, thereby warranting further attention from a psychometric perspective.

When considering the relationship between organizational culture and workplace outcomes, it is important to consider the psychometric properties of the instrument used to measure organizational culture, especially when considering the variety of instrumentation options available [1], [11], [12]. Jung et al.'s recent review of the psychometric properties of 48 organizational culture instruments noted that less than half (46%) of the instruments had published data demonstrating adequate internal consistencies. Additionally, only one in five (21%) instruments demonstrated adequate evidence for aggregating individual data to be representative of the organization as a whole [1], possibility resulting in erroneous assumptions about organizational-level culture where these measures are used. Lastly, Jung et al.'s review noted that only one in five (19%) of the examined instruments presented adequate evidence of the dimensionality of the instrument. The paucity of reliable, validated measures of organisational culture is particularly problematic given the applied context in which these measures are often used when facilitating cultural change [8], [9]. In this paper we examine the psychometric properties of a prominent diagnostic measure of organizational culture: the Organizational Culture Assessment Instrument (OCAI) [8], [9].

**Figure 1:**
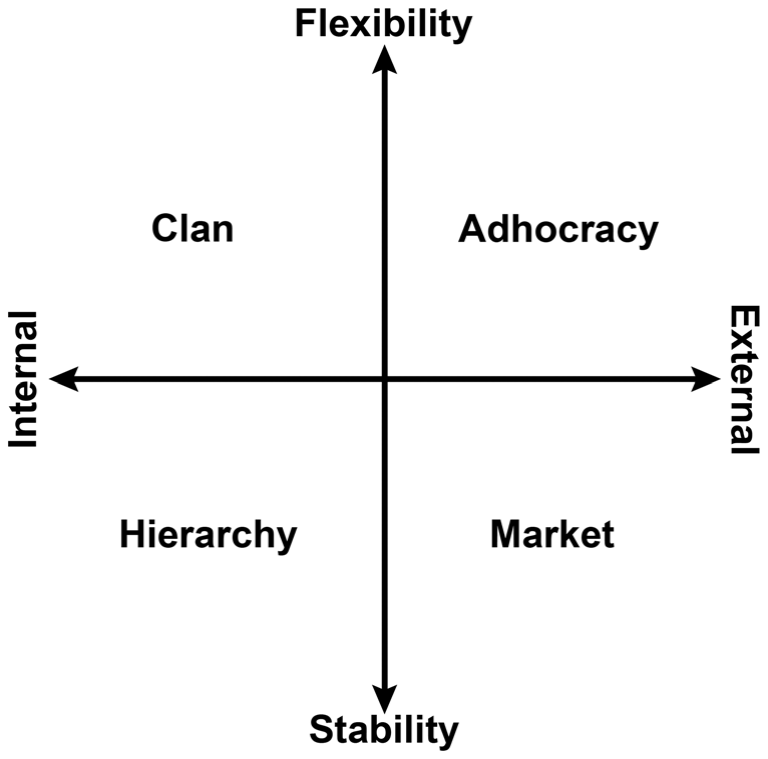
Factor structure of the OCAI reflective of the Competing Values Framework. Adapted from: Cameron, K. S., & Quinn, R. E. (2006). *Diagnosing and changing organizational culture: Based on the competing values framework* (Revised ed.). San Francisco, CA: Jossey-Bass.

The OCAI [8], [9] provides a diagnostic assessment of culture based on an examination of core values, shared assumptions, and common approaches to work. It is a classification approach to culture [12], and was designed to identify existing organizational culture as a prelude to cultural change. While acknowledging that the quantitative measurement of culture is controversial (e.g., [13]), Cameron and Quinn [9] claimed that the OCAI's use of quantitative data gathered from multiple individuals within the organization, tapping into the core values and related assumptions woven into the organization, can provide a realistic representation of its culture. The OCAI uses a four factor model to classify culture as falling along two bisecting continua; stability versus flexibility in work approaches, and internal versus external focus of the organization (see Figure 1) [8], [9].

The Clan culture archetype is reminiscent of Wallach's [14] supportive culture archetype, and is delineated by the flexibility and internal focus aspects of the OCAI's continua. It is considered to be representative of a family-style organization, wherein members of the organization are involved in decision making, and teamwork is an important aspect of work [8], [9].

The Adhocracy culture, which is delineated by the flexibility and external focus aspects of the bisecting continua of the OCAI, is based on innovation as a means of organizational functioning [8], [9]. One of the aspects of the Adhocracy is its emphasis on specialisation and rapid change within the organization; employees will often come together to work on specific projects and then disband at completion. This method of functioning is reminiscent of Martin and Meyerson's [15] ambiguity description of culture. However an organization with an Adhocracy culture is not limited by a lack of guidelines when approaching a task, and instead appears to be provoked into productivity when presented with a lack of boundaries.

The Hierarchy culture, delineated by the internal focus and stability aspects of the OCAI continua, is highly reminiscent of Wallach's [14] bureaucratic culture. It is concerned largely with stability in organizational functioning, and has clear guidelines regarding the manner in which organization should approach certain tasks [8], [9]. It is typified by a vertical approach to the levels in the organizational hierarchy, and focuses largely on smooth running efficiency.

Lastly, the Market culture is delineated by the external focus and stability aspects of the OCAI continua [8], [9]. This aspect of the OCAI is concerned largely with competitiveness and winning. The market culture is driven by the need to create transactions with external bodies as a means of gaining an advantage in their organizational niche.

Using the OCAI, these four factors provide the basis of cultural classification within the workplace [8], [9]. Additionally, the OCAI allows predictions to be made due to the process of reciprocal opposition [16], which in the context of this measure concern the factors diagonally opposite each other in Figure 1 (i.e., Clan and Market cultures, and Hierarchy and Adhocracy cultures). Nelson and Gopalan have previously noted that opposing clusters of values have been observed to carry inverted relationships with other outcome variables, and this notion has been applied to the OCAI factors' expected relationships with other organizational variables [8], [9]. The conflicting cultural characteristics inherent in each diagonally-opposed factor have been supported by a managerial-level variant of the OCAI framework [8], [9], as correlations between opposing factors were moderate-to-strong and negatively weighted. Therefore an important feature of the OCAI is not only that it describes organizational culture depending on alignments with the bisecting continua previously discussed, but that it also specifies the expected reciprocally-opposing pattern of relationships between culture factors and other organizational variables of interest. However, as Cameron and Quinn only cite their *exploratory* validation results regarding this property of the measure, the substantiation of these properties of the OCAI warrants further examination.

### Validation of the OCAI

Two previous studies have investigated the dimensionality and internal consistency of the OCAI [17], [18], with a third study validating these properties on a Korean translation of the OCAI [19]. Of note, all three studies employed confirmatory techniques to examine the factor structure of the OCAI, an advancement on the exploratory methodologies outlined by Cameron and Quinn [8], [9] in support of their instrument. As confirmatory factor analysis (CFA) is a theoretically-driven approach to model validation, thereby diluting the capitalisation on chance associated with exploratory techniques, it provides a much more rigorous form of model validation [20]. Relatedly CFA can account for measurement error during assessment of model adequacy, thereby providing a much finer-grained approach to model suitability testing compared to exploratory variants. We therefore view the results of CFAs conducted on the OCAI as a more rigorous, and therefore presumably more valid, estimation of the measure's properties.

Kalliath et al. [18] investigated the dimensionality and internal consistency properties of an early version of the OCAI [21], reporting excellent internal consistency indices (>.80 alpha; [22]) for each of the four factors. The authors also presented evidence of sufficient model fit for the OCAI and reported a range of significant and non-significant relationships between the two pairs of opposing factor dyads, providing mixed evidence for the notion of reciprocal opposition [16] underlying the instrument. A major limitation of this study was the use of a sample comprising managerial and supervisory staff members only. As managerial employees are sources and perpetuators of organizational culture [23], as demonstrated on the basis of their personalities and leadership styles [24], [25], sampling from this strata of the organization may produce different results compared to sampling from a wider range of employees. Further, no attempt was made to examine the validity of the instrument in relation to other workplace measures. In summary, Kalliath et al.'s [18] CFA of the OCAI instrument validated the four factor dimensionality and internal consistency of an early version of the measure. However, the non-representative sample and mixed support for the reciprocal opposition underlying the instrument is potentially problematic.

The second study of the psychometric properties of the OCAI, Helfrich et al.'s [17] CFA of the OCAI, overcame some of the issues in the earlier study by Kalliath et al. [18]. A larger sample (approximately 72000 participants) from a broader spectrum within the organizational hierarchy of the Veteran Health Administration was used. Zammuto and Krakower's [26] scale, an earlier version of the OCAI based on the same four archetype framework as Cameron & Quinn's [8], [9] later measure, was modified with two items (representative of the equivalent Market and Clan cultures) removed due to concerns regarding survey length, resulting in a total of 14 items indicative of the four domains of culture. While internal consistency of three of the four factors was satisfactory, the Hierarchical factor was found to have less than ideal reliability (*a* = .69). CFA findings were not supportive of the four factor model, with exploratory follow-up analyses extracting a two factor model consisting of the Entrepreneurial, Team, and Rational factors (akin to the Adhocracy, Clan, and Market factor respectively) loading on one latent factor, while three Hierarchical items loaded on a separate factor. While wording and scale changes in measurement used by this version of the instrument were presented by Helfrich et al. as possible factors influencing the unexpected two factor solution, it is concerning that one of the three CFAs conducted on the OCAI to date has demonstrated divergent dimensionality of the instrument.

Choi and colleagues' [19] examination of the validity of the Korean translation of the OCAI included both internal consistency and factorial validation analyses. The authors noted that Clan and Adhocracy factors had the highest internal consistency values, however they did not provide specific information on the Hierarchy and Market factors. While the chi-square results for model fit of the OCAI were significant, their data demonstrated good *NFI* and *RMSEA* values, thereby providing acceptable model fit for the OCAI upon acknowledging the probable sample-size bias in chi-square significance values noted in the literature [27]. A limitation of the study was the underpowered analysis, based on data from only 133 participants. Of additional interest to the current study are the correlations between factors reported by Choi et al., and their lack of concordance with the OCAI's purported reciprocally opposing relationships between diagonal factors. The correlations between the Clan/Market and Adhocracy/Hierarchy factors were all significant, strong (*r* = .89) and moderate (*r* = .52) respectively, and notably bearing positive coefficient directions. Cameron and Quinn [8], [9] have previously noted that diagonally opposing factors, such as Clan and Market, would be expected to have competing values and assumptions that would lead these cultural types to be in conflict with another. It is therefore contrary for Choi et al.'s results to have indicated strong positive relationships between theoretically polar factors. Adding to the limited consistency in OCAI validation presented by the studies of Kalliath et al. [18] and Helfrich et al. [17] prior, Choi and colleagues' evidence of the adequacy of the OCAI's factor structure was again restricted.

Further contradictions to the expected reciprocally-opposing pattern of relationships between culture factors of the OCAI and other organizational variables of interest have appeared in the literature. Hartnell, Ou, and Kinicki's [28] meta-analysis of the properties of the OCAI, focusing on the relationships between the culture factors and organizational effectiveness indicators, noted positive correlations with job satisfaction and Clan, Adhocracy, and Market culture. Market culture's positive correlation was similar in strength to that of Clan culture, which does not appear to be indicative of conflicting relationships between opposing factors on organizationally-relevant outcomes. It is therefore ambiguous as to whether there are broad oppositional qualities with specific organizational outcomes for each opposing culture dyad, or whether these oppositional qualities are only present at a comparatively local level. Hierarchy culture data was unfortunately not analysed in Hartnell and colleagues' study. It is therefore unclear whether the reciprocal opposition aspect of the OCAI is a broadly validated aspect of the measure, and would benefit from further scrutiny. Therefore further examination of the model properties of the OCAI is warranted in the pursuit of assessing the validity of the instrument.

### Significance of the Current Study

As a means of adding to and clarifying the existing literature on the psychometric properties of the OCAI [8], [9], the current study examines the factor structure and validity of the current version of the OCAI. Building on the broad approach to sampling across organizational strata by Helfrich et al. [17], the current study will also address employee perceptions of organizational culture from both an ideal and current culture approach similar to that seen in the Person-Organization (P-O) fit literature (e.g., [7]). Asking employees to provide details of their ideal and current organizational culture along the OCAI dimensions provides the basis for examining consistencies in culture conceptualisation across employee perspectives. This is one of the areas seemingly assumed but rarely tested according to Jung et al.'s [1] review of the culture instrumentation literature. None of the previously outlined CFA-based validation studies examined model invariability across the ideal and current organizational culture perspectives, thereby warranting its inclusion in the validity examination of the OCAI in this study. As Cameron and Quinn's [8], [9] OCAI asks participants to assess current and ideal preferences for culture (the equivalent of perceived organizational and individual preferences respectively), it is an oversight that the model's adequacy has not been tested across these two data perspectives. Therefore the following model validation aspects of OCAI are proposed for examination:

*1a*. Using ideal culture data, the OCAI will demonstrate adequate model fit criteria.

*1b*. Adequate internal consistency (*a*>.80) will be demonstrated for each factor based on ideal culture data.

*2a*. Using current organizational culture data, the OCAI will demonstrate adequate model fit criteria

*2b*. Adequate internal consistency (*a*>.80) will be demonstrated for each factor based on current organizational culture data.

3. The factor structures of the OCAI best-fitting to the data will be consistent across data perspectives.

Lastly, as a means of examining criterion validity, the OCAI will be examined in relation to job satisfaction. While previous studies have identified relationships between job satisfaction and organizational culture (e.g., [29], [30]–[34]), the current study will examine the reciprocally-opposing relationships in addition to the criterion validity links to job satisfaction. Despite Hartnell et al.'s [28] meta-analysis which noted positive correlations between job satisfaction and the Clan, Adhocracy, *and* Market culture factors, our predictions are based on the hypothesised reciprocally-opposing cultures (and prospectively, their ties to organizationally-relevant outcomes) between the Clan and Market factors. Thus, the following aspects of the model are expected to emerge:

*3a*. Using ideal culture data, significant positive relationships between the Clan culture predictor and job satisfaction will be present.

*3b*. Using current organizational culture data, significant positive relationships between the Clan culture predictor and job satisfaction will be present.

*4a*. Using ideal culture data, significant positive relationships between the Adhocracy culture predictor and job satisfaction will be present.

*4b*. Using current organizational culture data, significant positive relationships between the Adhocracy culture predictor and job satisfaction will be present.

*5a*. Using ideal culture data, significant negative relationships between the Hierarchy culture predictor and job satisfaction will be present.

*5b*. Using current organizational culture data, significant negative relationships between the Hierarchy culture predictor and job satisfaction will be present.

*6a*. Using ideal culture data, significant negative relationships between the Market culture predictor and job satisfaction will be present.

*6b*. Using current organizational culture data, significant negative relationships between the Market culture predictor and job satisfaction will be present.

## Methods

### Ethics Statement

This research was approved by Curtin University's Human Research Ethics Committee (Reference number HR63/2008).

### Design

The research design was single, cross-sectional study with organizational participant data gathered via an online survey.

### Participants

Participants were a convenience sample of public sector or private health employees from Western Australia, with 328 participants (male *N = *102, female *N* = 226) in total. Forty two participants were sourced from private healthcare, while the remaining 286 employees were participants from local government. Participants were aged between 18 and 73 years (*M* = 39.79 years, *SD* = 12.57), had occupational tenure of between 0.5 and 55 years (*M* = 11.00 years, *SD* = 11.26), and organizational tenure between 0.5 and 40 years (*M* = 4.21 years, *SD* = 6.90).

Of particular relevance to the current study's attempt to provide further information on the validity of the OCAI, it is noteworthy that previous studies using confirmatory factor analysis techniques on this instrument have sampled exclusively from populations within the United States and South Korea (e.g., [17], [19]). It is therefore of interest for further validation of the instrument that its factor structure and criterion validity are examined outside of these previously examined sample countries, as this would in-part provide evidence of cross-cultural validity.

#### Sample Size and Power

The sample of 328 participants meets the minimum power requirement of at least 5 to 10 times the amount of indicators in the CFA model [35] and the recommended 10∶1 ratio of cases to free parameters [27]. The ratio of 20 to 40 times the amount of cases to entered predictors ratio for HMRA [36]–[38] is also satisfied by the participant total.

### Measures

#### Culture

The four archetypical profiles of organizational culture were measured using the 24 item Organizational Culture Assessment Instrument (OCAI) [8], [9]. An example item from the Clan scale is “The organization is a very personal place. It is like an extended family. People seem to share a lot of themselves”. An example item from the Adhocracy scale is “The organization is a very dynamic and entrepreneurial place. People are willing to stick their necks out and take risks”. An example item from the Hierarchy scale is “The organization is a very controlled and structured place. Formal procedures generally govern what people do”. An example item from the Market scale is “The organization is very results-oriented. A major concern is with getting the job done. People are very competitive and achievement-oriented”. Participants were asked to respond to each item using a 5 point Likert scale (1 = strongly disagree, 5 = strongly agree). This change to the usual ipsative response format for the OCAI (in which participants distribute 100 points between 4 statements to indicate organizational relevance) was used to accommodate the on-line testing format, and in concordance with the past analyses of Kalliath et al. [18] and Helfrich et al. [17]. Participants were first asked to respond to the 24 items based on their perceptions of current organisational practises. They then responded to the 24 items again based on their ideal organisational practices. Scale reliability for each of the four archetypal profiles from the original measure has been demonstrated as sufficient, with Cronbach's α ranging from .71 to .80 [8], [9].

#### Job Satisfaction

Job satisfaction was measured using a 15 item instrument comprising an intrinsic and extrinsic subscale [39]. Example items assessed for satisfaction include “Your fellow workers” and “Your rate of pay” [39]. Items were scored on a seven point Likert-type scale, with a score of 1 indicating “I'm extremely dissatisfied” and 7 indicating “I'm extremely satisfied”. The global scale has previously demonstrated good internal reliability (Cronbach's α .80 to .91 across studies; [40]). The intrinsic subscale has similarly good reliability (Cronbach's α .84 to .88; [40]), with sufficient reliability for the extrinsic subscale (.76; [40]). Fields [40] noted that overall job satisfaction as measured by the scale correlated positively with psychological well-being, pay satisfaction, and perceptions of job control and competence. The scale correlated negatively with job control problems and job-based tension, supporting the validity of the measure. A total score of job satisfaction from the measure was used in the forthcoming analyses.

#### Demographic Variables

Age, gender, organizational tenure, and occupational tenure were measured using single items for prospective inclusion as control variables in the analysis. Age and tenure have been previously linked to organizational commitment [41]. Gender has been previously linked to differences in job satisfaction [42], [43].

### Procedure

Following Curtin University's Human Research and Ethics Committee approval, potential organizations for the study were contacted by email and phone call. Ten of 50 contacted local government organizations agreed to participate (20% participation rate), and one private healthcare organization agreed to participate. Organizations distributed an email to staff members offering them the opportunity to participate in the study, with employees clicking on a link to an online questionnaire if they wished to participate. Participants were offered the chance to enter a prize draw to win a gift voucher if they completed the survey, a strategy that increases both response and completion rates in online surveys [44]. The data sets from each organization were combined to create a complete data file containing the information of all sampled organizations.

## Results

### Missing Data Analysis and Control Variables

A missing values analysis was conducted using SPSS 19.0. None of the items had missing data in excess of 9%. Little's MCAR test was significant, *χ* ^2^_(26006, N = 328)_ = 26420.66, *p* = .035, indicating the data was not missing completely at random MCAR. Examination of the follow-up Bonferroni-corrected *t*-tests revealed no statistically significant *p* values, therefore the missing data was considered missing at random, and was replaced using multiple imputation techniques.

Age, gender, organizational tenure, and occupational tenure were not significantly related with job satisfaction and were not included as control variables in the upcoming MLM analyses.

### Ideal Culture CFA

All inferential tests were tested using an α level of .05 unless otherwise indicated. CFA was conducted to validate the model fit criteria of the OCAI [8], [9]. All latent factors were permitted to correlate with each other during the model assessment, due to the unclear nature of reciprocal opposition noted in Kalliath et al.'s [18] prior CFA. The CFA analyses were repeated for both the ideal culture and current organizational culture data to assess similarity of factor structures. LISREL (Version 8.80 for Windows) was employed during CFA testing. All assumptions were tested and met prior to conducting the analysis.

The first CFA was conducted to determine whether the ideal culture (IC) data conformed to the hypothesised OCAI model [8], [9]. A unidimensional model, with all OCAI indicators loading onto a single factor representing IC, was first tested for use as a baseline against which to assess the fit of the four factor model. The unidimensional model had poor fit (see Table 1). The *SRMR*, *CFI*, and *RMSEA* coefficients were all outside the recommended statistical cut-offs indicative of adequate model fit [20], [27]; SRMR≤.08, CFI≥.95, and .05≤RMSEA≤.08 for reasonable fit (inclusive of the consideration of the 90% confidence intervals).

**Table 1:** Comparisons of Fit Indices between the Unidimensional and Hypothesised Models of Ideal Culture.

	*df*	χ^2^	*p*	*SRMR* ^a^	*CFI* ^b^	*RMSEA* ^c^	90% *CI* ^d^
Uni^e^	252	1293.66	.001	.11	.71	.14	.13–.15
Four Factor	246	759.00	.001	.09	.89	.09	.08–.09
Revised Model	246	725.40	.001	.08	.91	.08	.07–.08
Δ Uni^e^-Revised	6	568.26	.001				

*Note*. ^a^ Standardised Root Mean Square Residual. ^b^ Comparative Fit Index. ^c^ Root Mean Square Error of Approximation. ^d^ 90% Confidence Interval for RMSEA. ^e^ Unidimensional.

The following CFA tested the four factor model presented by Cameron and Quinn [8], [9]. The preliminary CFA for the four factor IC data appeared to provide a near-acceptable model fit (see Table 1). However, the *CFI* was below the minimum recommended cut-off value [27], and *RMSEA* and its 90% confidence intervals had an upper range in excess of .08, therefore it was not considered a good model fit.

A combined statistical and theory-driven approach to model reassessment was undertaken. LISREL's reported modification indices suggested that the second item of the Hierarchy subscale should be remapped to the Clan latent factor. The item “The leadership practices in the organization are generally considered to exemplify *coordinating, organizing*, or smooth-running efficiency” (emphasis authors' own) [9] could be viably interpreted as belonging to the Clan culture. Clan culture was typified by consensus driven practices and being an ‘extended family’ in terms of its organizing behaviours. Therefore this item seemed sensible to include as an indicator of the Clan factor. This was further substantiated when it was noted that the Clan culture factor is not reciprocally opposed to the Hierarchy culture factor in the Competing Values Framework. A revised model was reassessed in LISREL, the fit indices of which are reported in Table 1.

**Figure 2:**
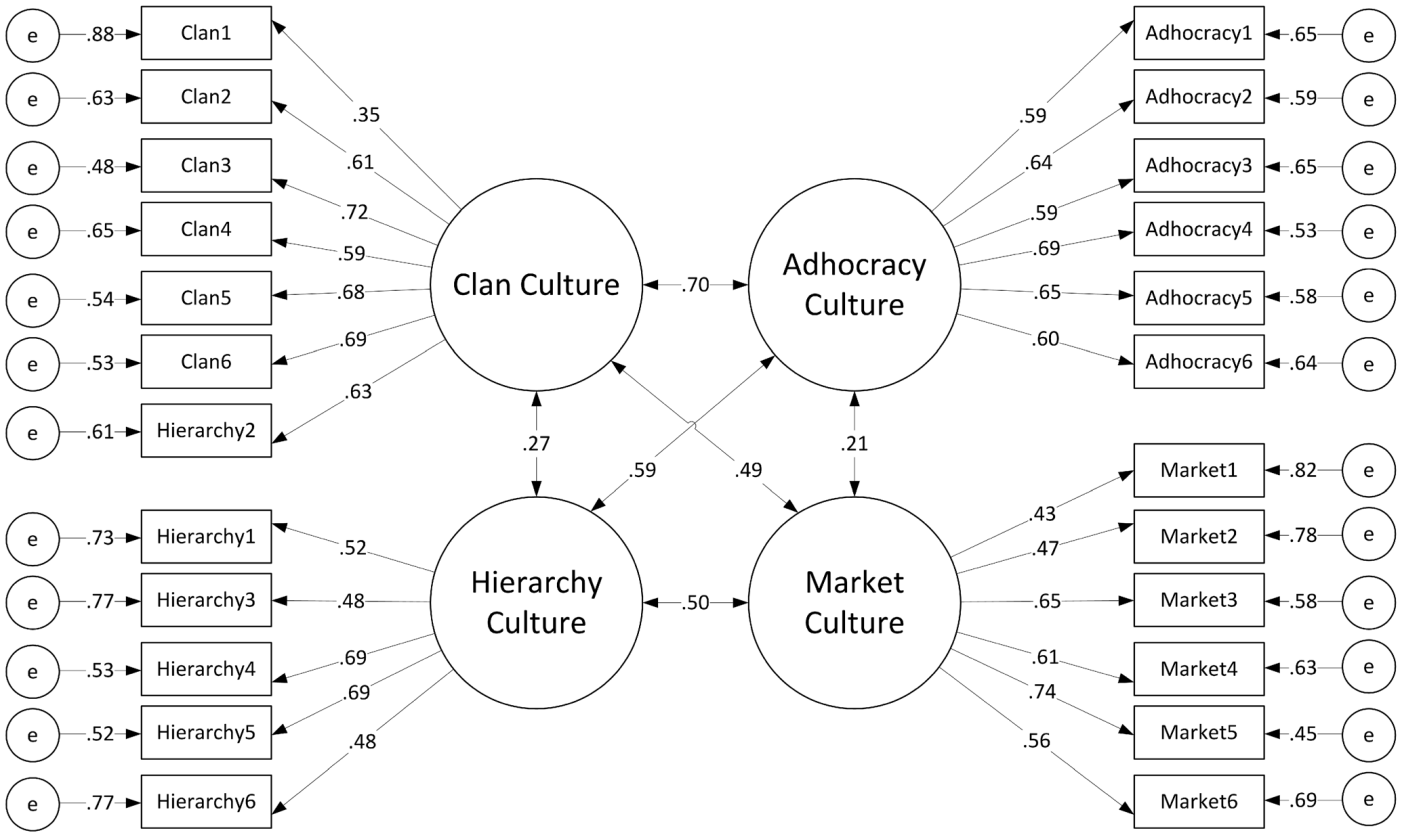
CFA results for revised four factor OCAI model of ideal organizational culture. Standardised fit indices and error terms of the revised four factor model of ideal organizational culture.

The revised four factor model of culture for the IC data had acceptable levels of model fit, as presented in Figure 2. There was a significant difference in model fit between the unidimensional model and the revised four factor model (see Table 1). Means, standard deviations, and reliabilities of the revised four factor model for individual preferences are presented in Table 2.

**Table 2:** Means and Standard Deviations of Ideal Culture Factors.

	*M*	*SD*	Minimum	Maximum	α Reliability
Clan Culture	4.15	.47	2.00	5.00	.80
Adhocracy Culture	3.70	.60	1.67	5.00	.79
Hierarchy Culture	3.08	.65	1.67	5.00	.69
Market Culture	3.82	.52	2.00	5.00	.75

### Current Culture CFA

The second CFA examined current culture (CC) for the four culture factors. The unidimensional solution, with all indicators loading onto a single factor representative of organizational culture as a whole, was not considered a good fit (see Table 3).

**Table 3:** Comparisons of Fit Indices between the Unidimensional and Hypothesised Models of Current Organizational Culture.

	*df*	χ^2^	*p*	*SRMR* ^a^	*CFI* ^b^	*RMSEA* ^c^	90% *CI* ^d^
Uni^e^	252	1655.60	.001	.14	.84	.13	.12–.14
Four Factor	246	739.68	.001	.08	.94	.08	.07–.09
Revised Model	246	698.56	.001	.07	.95	.07	.07–.08
Δ Uni^e^-Revised	6	957.04	.001				

*Note*. ^a^ Standardised Root Mean Square Residual. ^b^ Comparative Fit Index. ^c^ Root Mean Square Error of Approximation. ^d^ 90% Confidence Interval for RMSEA. ^e^ Unidimensional.

**Figure 3:**
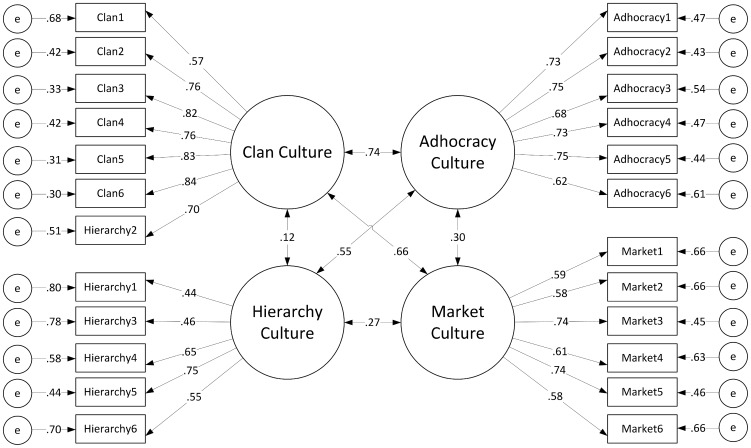
CFA results for revised four factor OCAI model of current organizational culture. Standardised fit indices and error terms of the revised four factor model of current organizational culture.

Following the unidimensional model, the hypothesised four factor model of culture was tested and produced close-to-acceptable indicators of model fit (see Table 3). LISREL modification indices again suggested that the Hierarchy item “The leadership practices in the organization are generally considered to exemplify coordinating, organizing, or smooth-running efficiency” should be remapped onto the Clan culture factor. The revised model with this item loading on the Clan culture had better model fit than the original four factor model (see Table 3 and Figure 3). There was a significant difference in model fit between the univariate model and the revised four factor model (see Table 3). Means, standard deviations, and Cronbach's [22] alpha reliability coefficients of the revised four factor model are presented in Table 4.

**Table 4:** Means and Standard Deviations of Current Organizational Culture Factors.

	*M*	*SD*	Minimum	Maximum	α Reliability
Clan Culture	3.21	.95	1.00	5.00	.90
Adhocracy Culture	2.79	.81	1.00	4.83	.86
Hierarchy Culture	2.85	.73	1.17	5.00	.70
Market Culture	3.70	.65	1.60	5.00	.80

Model aspects 1a and 2a, predicting good model fit for the OCAI for IC and CC data, were broadly supported by the CFA findings. Only minor model modification (the one item across the two models) was required. Model aspects 1b and 2b, predicting adequate internal consistencies for all latent factors, was broadly supported, however not all alpha values were >.80 as expected in our predictions of the instrument's qualities. Given that the internal consistency coefficients were generally >.70 (noting that Hierarchy culture from the IC model was *a* = .69, the lowest calculated coefficient), the internal consistency results for the OCAI appeared to be representative of adequate internal consistency. The third model aspect, which predicted model structure consistency between ideal and current culture data, was also supported by the results.

It should be noted that chi-square values were significant for all tested models, which may be indicative of poor model fit. However, given the chi-square test's sensitivity to smaller deviations as sample size increases [27], thereby inflating the probability of a significant chi-square result, the approach to evaluating model fit was based on the pattern of results across the array of fit indices. As the fit indices outside of the chi-square coefficient indicated acceptable model fit in both of the revised four-factor models for the instrument, we considered this to be sufficient evidence for structural acceptability.

### Relationship between Culture and Job Satisfaction

To examine the relationship between ideal or current culture and job satisfaction, multilevel modelling (MLM) was conducted. As MLM can account for intra-organizational variance in job satisfaction, thereby providing a clearer representation of its relationship with the OCAI factors, it was the preferred analysis in comparison to traditional multiple regression analysis. Two analyses were conducted; one for the IC data as predictors of job satisfaction, and one for CC data predicting job satisfaction as a parallel analysis.

#### MLM for IC Predicting Job Satisfaction

MLM requires at least 10 cases per organisation [45]. One case was removed as not meeting this assumption. Three further cases were removed as they were univariate outliers. Four cases in total were therefore removed from the upcoming analyses (1.22%), leaving 324 valid participants. All remaining assumptions underlying MLM were met. The null model, loading the workplace origin variable as a source of between groups variance in job satisfaction, is summarised in Table 5. Maximum likelihood was used as the method of estimation, with the null model's -2 Log Likelihood (-2LL) reported as −392.36.

**Table 5:** Null Model of Organizational Origin Variability in Accounting for Job Satisfaction with Ideal Culture Data (*N* = 324).

	*E ^a^*	*SE*	*df*	*t*	*Wald Z*	*p*
Fixed Effects						
Intercept	1.36	.012	8.82	117.64		.001^***^
Random Effects						
Residual	.02	.001			12.53	.001^***^
Intercept (Origin)	.00	.001			1.22	.112^b^

*Note*. ^a^ Estimate, ^b^ One-tailed *p* value, ^***^ *p*<.001.

The null model indicated that there was not a significant proportion of job satisfaction attributed to inter-workplace differences (*p* = .112, one-tailed). This was further reinforced by the small Intra-class Correlation Coefficient [ICC] (.042), or approximately 4.2% of the variance in Job Satisfaction being attributable to inter-organizational differences.

The four culture predictors were entered as fixed effect indicators of Job Satisfaction. The results indicated a small difference, −2LL = −400.39, Δ-2LL = 8.03, *p* = .005, and is summarised in Table 6.

**Table 6:** Model of Culture Indicators Predicting Job Satisfaction for Ideal Culture Data (*N* = 324).

	*E ^a^*	*SE*	*df*	*t*	*Wald Z*	*p*
Fixed Effects						
Intercept	1.30	.097	321.96	13.43		.001^***^
Clan	.09	.058	323.51	1.56		.120
Adhocracy	−.01	.050	323.12	−.29		.771
Hierarchy	.05	.046	323.02	1.07		.287
Market	−.09	.049	318.90	−1.81		.072
Random Effects						
Residual	.02	.001			12.52	.001^***^
Intercept (Origin)	.00	.001			1.02	.153^b^

*Note*. ^a^ Estimate, ^b^ One-tailed *p* value, ^***^ *p*<.001.

A small difference in explained variance between the null model and the experimental model was noted, Δ*R* ^2^ = .019, or 1.9%. However this difference in variance was marginal, and none of the culture predictors were significant indicators of Job Satisfaction. Therefore, none of the four ideal culture indicators of the OCAI were significantly related to Job Satisfaction, providing no support for the expected pattern of results suggested by aspects 3a, 4a, 5a, and 6a.

#### MLM for CC Predicting Job Satisfaction

One case was removed due to being a multivariate outlier, leaving 326 valid participants after also removing the lone participant from one organization. All other assumptions underlying MLM were met. Similar to the IC MLM, the null model was first established, −2 Log Likelihood = −396.06 (see Table 7).

**Table 7:** Null Model of Organizational Origin Variability in Accounting for Job Satisfaction with Current Culture Data (*N* = 326).

	*E ^a^*	*SE*	*df*	*t*	*Wald Z*	*p*
Fixed Effects						
Intercept	1.36	.011	8.73	119.21		.001^***^
Random Effects						
Residual	.02	.001			12.57	.001^***^
Intercept (Origin)	.00	.001			1.20	.116^b^

*Note*. ^a^ Estimate, ^b^ One-tailed *p* value, ^***^ *p*<.001.

The null model again indicated that there was no significant proportion of Job Satisfaction's variance explained by workplace differences (*p* = .116). The *ICC* was additionally low, *ICC* = .040, or approximately 4.0% of the variance in Job Satisfaction was attributable to workplace differences.

The four CC data indicators were entered as fixed effects when predicting Job Satisfaction, −2 Log Likelihood = −564.03, Δ-2LL = 167.97, *p*<.001. The predictor coefficients and their significance are presented in Table 8.

**Table 8:** Model of Culture Indicators Predicting Job Satisfaction for Current Culture Data (*N* = 326).

	*E ^a^*	*SE*	*df*	*t*	*Wald Z*	*p*
Fixed Effects						
Intercept	1.06	.061	305.46	17.42		.001^***^
Clan	.24	.033	289.61	7.12		.001^***^
Adhocracy	.04	.011	307.89	3.27		.001^**^
Hierarchy	.00	.033	326.00	.05		.963
Market	−.11	.033	325.49	−3.32		.001^**^
Random Effects						
Residual	.01	.001			12.60	.001^***^
Intercept (Origin)	.00	.000			.110	.456^b^

*Note*. ^a^ Estimate, ^b^ One-tailed *p* value, ^**^ *p*<.01, ^***^ *p*<.001.

Approximately 38.8% of the variability in Job Satisfaction scores was explained by the predictors in unison (Δ*R* ^2^ = .388). CC Clan and Adhocracy had significant positive coefficients when explaining Job Satisfaction, while Market culture preferences were negatively related to Job Satisfaction in this analysis, providing partial support for the expected links between the CC culture factors and Job Satisfaction (model aspect 5b was not supported).

## Discussion

The findings of the current study indicated that the four factor model underlying the OCAI [8], [9] is broadly supported by the confirmatory factor analyses. The same four factor model demonstrating good model fit criteria was found for both IC and CC data. However, one item from the Hierarchy factor provided better model fit when applied to the Clan culture factor (for both IC and CC models), thereby departing from the OCAI as presented by Cameron and Quinn [8], [9]. Each factor had adequate internal consistency in both IC and CC models.

The results of the current study are supportive of Kalliath et al.'s [18] and Choi et al.'s [19] CFA results, demonstrating a four factor model underlying the OCAI. Measures of internal consistency were generally higher than those noted by Kalliath et al. [18] for the CC data model, and were comparable with those of Kalliath et al. for the IC data model.

Additionally, the current study validated the four factor structure of the model from two different perspectives; ‘ideal’ culture and current culture perceptions. The OCAI [8], [9] asks for evaluations along both perspectives during organizational culture change assessment, and evidence of invariance of the factor structure across perspectives was not provided in the previous CFA studies by Kalliath et al. [18], Helfrich et al. [17], and Choi et al. [19]. Therefore this is a notable addition to the validation of the instrument. However, this consistency could be due to the data being derived from the same source (the employee), and therefore the same perceptual schema of culture being applied identically in both situations. This common-method bias is a possible explanation for the concordant CFA results. Further confirmation of the structural validity of the OCAI from varying data perspectives will be necessary in future studies.

The differences in the construction of the Hierarchy latent factor in the current study may be due to previous structural analyses of the OCAI using multi-dimensional scaling as an analysis technique [(e.g., [8], [9]), which is an exploratory form of structural analysis. Alternatively, it may be due to the differences in wording between the contemporary OCAI and Quinn and Spreitzer's [21] earlier edition used by Kalliath et al.'s [18] CFA validation. The contemporary OCAI uses a two sentence statement per culture item, potentially allowing methodological issues such as asking double-barrelled questions to influence the integrity of the derived results. Why this has not influenced the other three factors is unclear, however. Alternatively, the reappropriated Hierarchy indicator may be a sample specific anomaly. The sample used in these analyses was sourced from local government and private healthcare settings. It may be possible that the Hierarchy culture was ambiguously represented at these specific workplaces, such that the employees undertaking culture assessment were unsure about whether their culture reflected a hierarchy-based culture or not. This lack of clarity, perhaps in the form of contradictory application of some aspects of the hierarchy-based culture and dismissal of others akin to Martin and Meyerson's [15] ambiguity conceptualization of culture, could contribute to this result. Future confirmatory factor analysis examination of the OCAI should investigate whether the reappropriated indicator is replicable in improving model fit.

Despite an item reappropriation during both CFAs, *thematically* there was little deviation from Cameron and Quinn's [8], [9] conceptualization of the CVF as measured by the OCAI, and of the findings presented by Kalliath et al. [18] and Choi et al. [19]. The item swapped was arguably consistent with the definition of the Clan culture due to its focus on coordinating and organizing among employees, which can be indicative of a consensus driven approach to culture typical of the Clan culture. Therefore, while there was only partial support of the OCAI model fit predictions due to the item reappropriation to achieve acceptable model fit, the model was arguably supportive of the measurement intent underlying the OCAI.

The findings regarding the relationship between the OCAI and job satisfaction were mixed. Ideal culture factors were not significant predictors of job satisfaction. This was surprising given previous literature demonstrating the linkages between culture and job satisfaction [19]. However, Hofstede [2] has previously noted that organizational culture is meaningful at the organizational level, not at the individual level. Therefore individual preferences for culture, which according to Hofstede is a strictly organizational characteristic, may expectedly produce non-significant relationships with an outcome such as Job Satisfaction. This is compounded by the series of significant relationships with Job Satisfaction the current organizational culture data produced. As the deployed OCAI asked individuals about their ideal organizational culture, the use of this data may be limited to interactive usage with current organizational culture perceptions, and may not demonstrate main effects in isolation.

In contrast, the current organizational culture data indicated that the Clan, Adhocracy, and Market factors were significant predictors of job satisfaction. While prior meta-analytic results by Hartnell et al. [28] provided evidence of small to moderate positive correlations between Clan, Adhocracy, Market cultures and job satisfaction, it is interesting to note that Market culture had a negative coefficient direction in the current study. These results mirror correlational findings by Lovas [46], who similarly measured public sector employees and the ties between the OCAI factors and job satisfaction. Lovas found significant relationships between the Clan, Adhocracy, and Market factors bearing the same coefficient directions as the current study, and similarly did not find a significant predictor effect for the Hierarchy factor. It is worthwhile to note however that while the MLM results demonstrated divergent ties to job satisfaction between theoretically opposing culture factors, the results of the CFAs did not seem to replicate this strong separation between factors. Figures 2 and 3 both demonstrate standardised coefficients with greater magnitudes for diagonally opposing factors (e.g., Clan and Market) versus adjacent factors (e.g., Clan and Hierarchy). This was a surprising result given the theoretical polarity the diagonal factors are designed to reflect, which would have presumably lead to deflated relationships between opposing factors, and comparatively stronger relationships between adjacent culture factors. Further investigation of the relationships between the OCAI factors at a structural level would be beneficial to examine in future research as a means of eliciting further detail on the suggested oppositional nature of the diagonal factors.

The absence of a relationship between the Hierarchy culture factor and job satisfaction is interesting to consider based on the prior discussion of the structural problems associated with this latent factor. Berson et al. [47] presented findings that indicated bureaucratic organizational culture preferences were significantly related to job satisfaction and organizational efficiency. As previously inferred, a possible degree of ambiguity regarding the manner in which the organization reacts in accordance to the hierarchy cultural archetype [15] could be diminishing any inferences made. Additionally, a sample-specific anomaly may have influenced the MLM findings regarding the association of Hierarchy culture and Job Satisfaction. Considering that the study sampled public sector and private healthcare employees, there may be a lack of influence of ‘Hierarchy’ factors due to heavily standardised methods of work within the organizations. Given that these cultural aspects may be taken for granted within these organizations, and may be embedded within employee assumptions, attributing any kind of influence of Hierarchy on job satisfaction may be difficult due to reasons of non-saliency. This may also explain in part the previously noted similarity with Lovas' [46] findings. Despite the incongruence of the Hierarchy culture findings with previous literature, the remaining significant findings support the hypothesised relationships between OCAI and job satisfaction.

Of note regarding the coefficient directions for the significant predictors of job satisfaction from the CC data are their opposing directions, conducive to the underlying notion of reciprocal opposition intended by Cameron and Quinn [8], [9] in their design of the OCAI. While previous meta-analytic results by Hartnell et al. [28] found consistent coefficient directions for the expectedly opposing Clan and Market factors, the current study did not. A possible explanation of this result is the ‘wider net’ being cast by Hartnell and colleagues in the literature search for their meta-analysis, as they were not solely using findings provided by previous studies that had used the OCAI. Instead, the authors chose culture-related findings for inclusion also on the basis of thematic-overlap with the factors of the OCAI, thereby prospectively muddying the reported relationships on the basis of subjectively-inferred overlap with the OCAI factors. In summary, the findings of the current study supported the inferred reciprocally-opposing relationships between the factors presented by Cameron and Quinn [8], [9].

### 

#### Limitations and Recommendations for Future Research

The sample for this study was exclusively recruited from the government and health care sector. In addition to the noted saliency limitations that may have influenced the Hierarchy culture factor, the composition of the sample may also account for the negative relationship found between CC Market culture and Job Satisfaction. Market culture may not necessarily be ‘bad’; it may just be considered unfavourable by the participants who took part in the study. Health care and public sector organisations may not be favourable domains to find a preference for hard-driving competitiveness, or sheer concern with profitability. For example, the influence of universal health care systems on the culture of a health care organization, specifically the tax-based funding inherent in Australia's healthcare system, may hypothetically diminish competitive/profitability culture alignment. Similarly, Australian government employees are working in a context where competitiveness and profitability are largely not of concern. Preferences for Market culture may not be negatively related to job satisfaction within alternative employment settings.

In addition to broader occupational sampling, further examination of the structure of the Hierarchy factor in the contemporary OCAI is warranted [8], [9]. Unaddressed by the current study, but noted during the testing process, are the length of the statements used by Cameron and Quinn in their description of cultural archetypes. Many of the items are double-barrelled, presenting the participant with multiple incidences of organizational behaviour representative of culture that they may agree with to varying degrees. Revising these lengthy statements may assist in improving the face validity of the instrument. Similarly, further evidence of discriminant and convergent validity of the OCAI beyond that reported by the instrument's authors [8], [9] would further reinforce the integrity of the instrument. The aforementioned areas provide valuable paths of future inquiry.

Furthermore, the results of the current study are based on an Australian sample. As previously noted, further validation of the OCAI outside of the primarily United States/South Korean samples used to date in confirmatory model validation studies is warranted to examine the cross-cultural validity of the measure. While the current study is a further step in validating the OCAI's structure in a country not previously examined, future research using a multi-nation sample could provide greater evidence of the structural robustness of the measure across cultures.

#### Conclusion

The findings from this research provide further evidence of the psychometric properties of the OCAI and the validity of the instrument as a viable method of assessing organizational culture. The four factors of culture (Clan, Adhocracy, Hierarchy, and Market) were all successfully validated as part of the larger model, albeit with adjustments to the Hierarchy factor. The instrument also demonstrated predictive validity due to its array of significant relationships with job satisfaction, a common indicator of organizational health. However, while the relationships between CC factors and job satisfaction were in partial support of the expected series of results, the IC factors were all redundant predictors of job satisfaction. While Jung et al. [1] previous highlighted limited evidence of the psychometric suitability of various culture assessment instruments, the OCAI appears to be a broadly sound instrument for diagnostic culture research as far as factor structure is considered, however it demonstrated mixed criterion validity in the current study.
